# Atypical presentation of esophageal Dieulafoy: diagnostic and therapeutic challenges in end stage achalasia

**DOI:** 10.1093/jscr/rjaf941

**Published:** 2025-12-04

**Authors:** Yosor Fiesal, Gad Aptilon, Amit Katz, Yoram Kluger, Leonid Drober

**Affiliations:** Department of General Surgery, Rambam Medical Center and the Bruce Rappaport Faculty of Medicine, Technion – Institute of Technology, Haifa, Israel; Department of General Surgery, Rambam Medical Center and the Bruce Rappaport Faculty of Medicine, Technion – Institute of Technology, Haifa, Israel; Department of Thoracic Surgery, Rambam Medical Center and the Bruce Rappaport Faculty of Medicine, Technion – Institute of Technology, Haifa, Israel; Department of General Surgery, Rambam Medical Center and the Bruce Rappaport Faculty of Medicine, Technion – Institute of Technology, Haifa, Israel; Department of General Surgery, Rambam Medical Center and the Bruce Rappaport Faculty of Medicine, Technion – Institute of Technology, Haifa, Israel

**Keywords:** achalasia, Dieulafoy, esophagus, vascular anomaly, gastrointestinal bleed

## Abstract

Dieulafoy lesions are rare vascular anomalies that can cause life-threatening gastrointestinal bleeding, most commonly in the stomach. Esophageal Dieulafoy lesions are exceptionally uncommon, particularly in patients with end-stage achalasia. We report a 46-year-old male with a history of achalasia and prior Heller myotomy who presented with acute hematemesis and hemorrhagic shock. Initial endoscopic and angiographic interventions failed to localize or control the bleeding. Computed tomography angiography revealed active bleeding in the distal esophagus. Surgical exploration identified a Dieulafoy lesion on the right lateral wall of the distal esophagus, which was successfully oversewn. Due to a severely dilated, nonfunctional esophagus, esophagectomy was performed, followed by delayed substernal gastric pull-up and cervical esophagogastrostomy. The postoperative course was complicated by acute respiratory distress syndrome and transient vocal cord paralysis, but the patient ultimately recovered well. This case illustrates the diagnostic and therapeutic challenges of esophageal Dieulafoy lesions in the context of end-stage achalasia. Early recognition, multidisciplinary management, and staged surgical reconstruction are critical for optimal outcomes in such complex presentations.

## Introduction

Dieulafoy lesions are rare but potentially life-threatening vascular anomalies of the gastrointestinal (GI) tract. They are characterized by large-caliber, aberrant submucosal arteries that protrude through small mucosal defects, leading to sudden, massive GI bleeding [1]. These lesions most commonly occur in the proximal stomach, particularly along the lesser curvature within 6 cm of the gastroesophageal junction. However, they can also be found throughout the GI tract, including the duodenum, colon, rectum, and, more rarely, the esophagus or small bowel [2–4].

A Dieulafoy lesion consists of abnormally large and tortuous artery, that erodes through the overlying mucosa, resulting in vessel exposure and a high risk of severe bleeding [5]. The adjacent mucosa usually appears unremarkable, complicating endoscopic identification, particularly in the absence of active bleeding.

Clinically, Dieulafoy lesions account for ~1%–2% of all cases of acute GI hemorrhage. They often present with painless, recurrent, and sometimes life-threatening bleeding. Endoscopy is both the primary diagnostic tool and the first-line treatment approach. Endoscopic hemostatic methods—including clipping, injection therapy, and thermal coagulation—have demonstrated high success rates in achieving bleeding control [6]. Surgical or angiographic interventions are generally reserved for cases where endoscopic management fails [7].

Surgical intervention is considered only when endoscopic therapies and, when available, angiographic interventions fail to control bleeding, or if the lesion is not endoscopically accessible. Surgical procedures may involve wedge resection of the affected segment of the stomach or intestine containing the lesion. Alternatively, if the bleeding site is accurately identified during surgery, direct oversewing of the exposed vessel may be performed. Minimally invasive laparoscopic techniques have also been described and are increasingly preferred due to their lower morbidity compared to traditional open surgery [7].

Reporting cases of Dieulafoy lesions in atypical locations—such as the esophagus—is crucial to raising clinical awareness and improving prompt diagnosis and management. Given the lesion’s potential to cause severe, rapidly progressive bleeding, early recognition is essential. Here, we present a rare case of a Dieulafoy lesion at the distal esophagus, highlighting diagnostic challenges and successful surgical treatment.

## Case report

**Figure 1:**
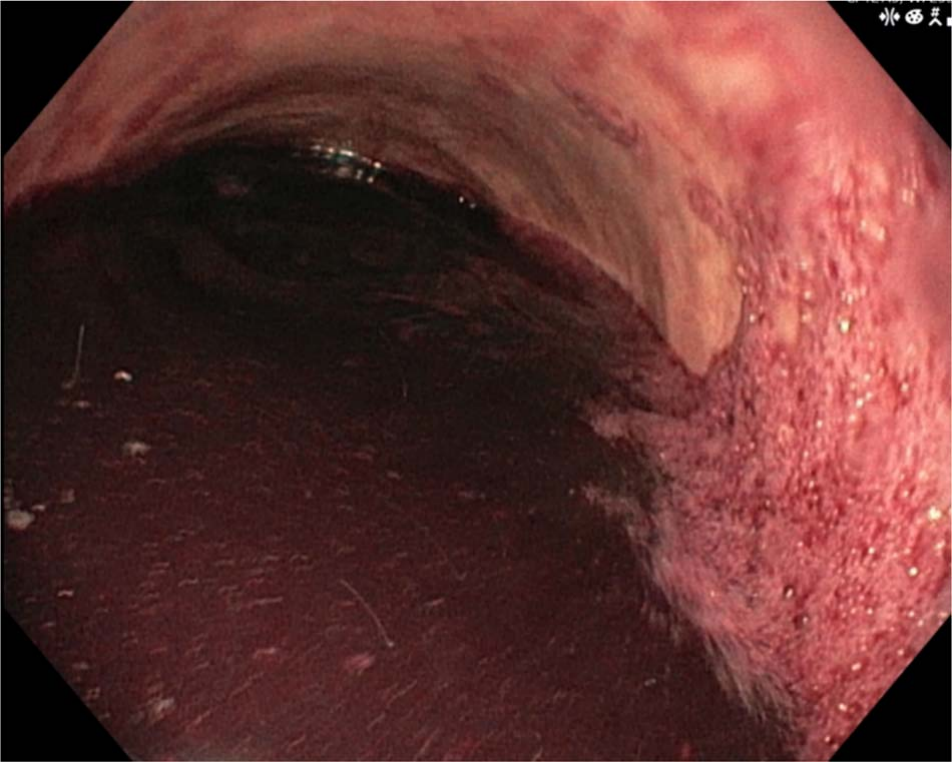
Demonstrates the gastroscopy findings, showing the stomach filled with blood.

**Figure 2:**
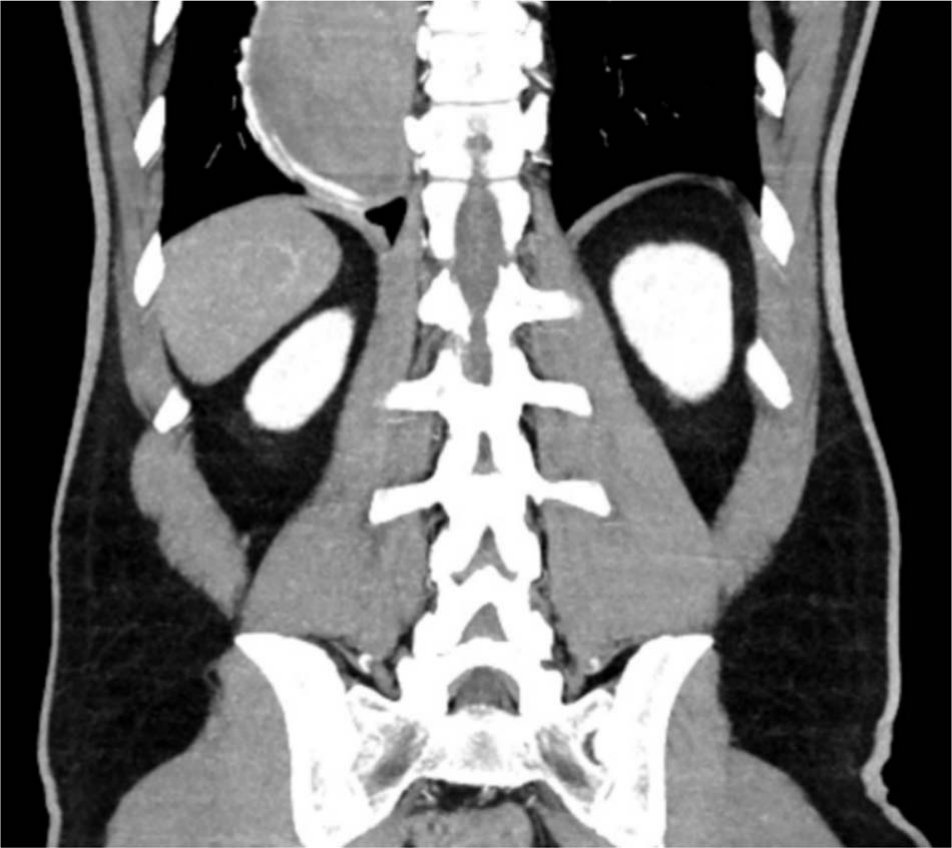
CT scan showing a markedly dilated esophagus with contrast blush in the distal third.

A 46-year-old male with a history of achalasia, for which he had undergone Heller myotomy with Nissen fundoplication in 2015, presented to the emergency department with a 2-day history of weakness and hematemesis. On admission, laboratory investigations revealed a significant drop in hemoglobin to 5 g/dl. Vital signs demonstrated tachycardia (122 beats per minute) and hypotension (70/50 mmHg), consistent with hemorrhagic shock. Initial management included aggressive resuscitation. Emergent gastroscopy was performed, which revealed the stomach filled with blood but failed to identify a clear bleeding source (Fig. 1). The patient remained hemodynamically unstable, necessitating transfusion of additional blood products. A computed tomography angiography was subsequently performed, revealing a markedly dilated, sigmoid-shaped esophagus, likely a sequela of end-stage achalasia. Furthermore, a contrast blush in the region of the distal esophagus, consistent with active ongoing bleeding (Fig. 2). An initial attempt was made to control the bleeding through angioembolization; however, this attempt was unsuccessful.

Following this, the patient was admitted to the surgical intensive care unit, where he required intubation and ongoing transfusion of blood products, totaling nine units of packed red blood cells and three units of fresh frozen plasma. Due to persistent hemodynamic instability and a strong suspicion that the source of bleeding resided in the distal esophagus or proximal stomach, a multidisciplinary meeting decided to proceed with surgical intervention.

**Figure 3:**
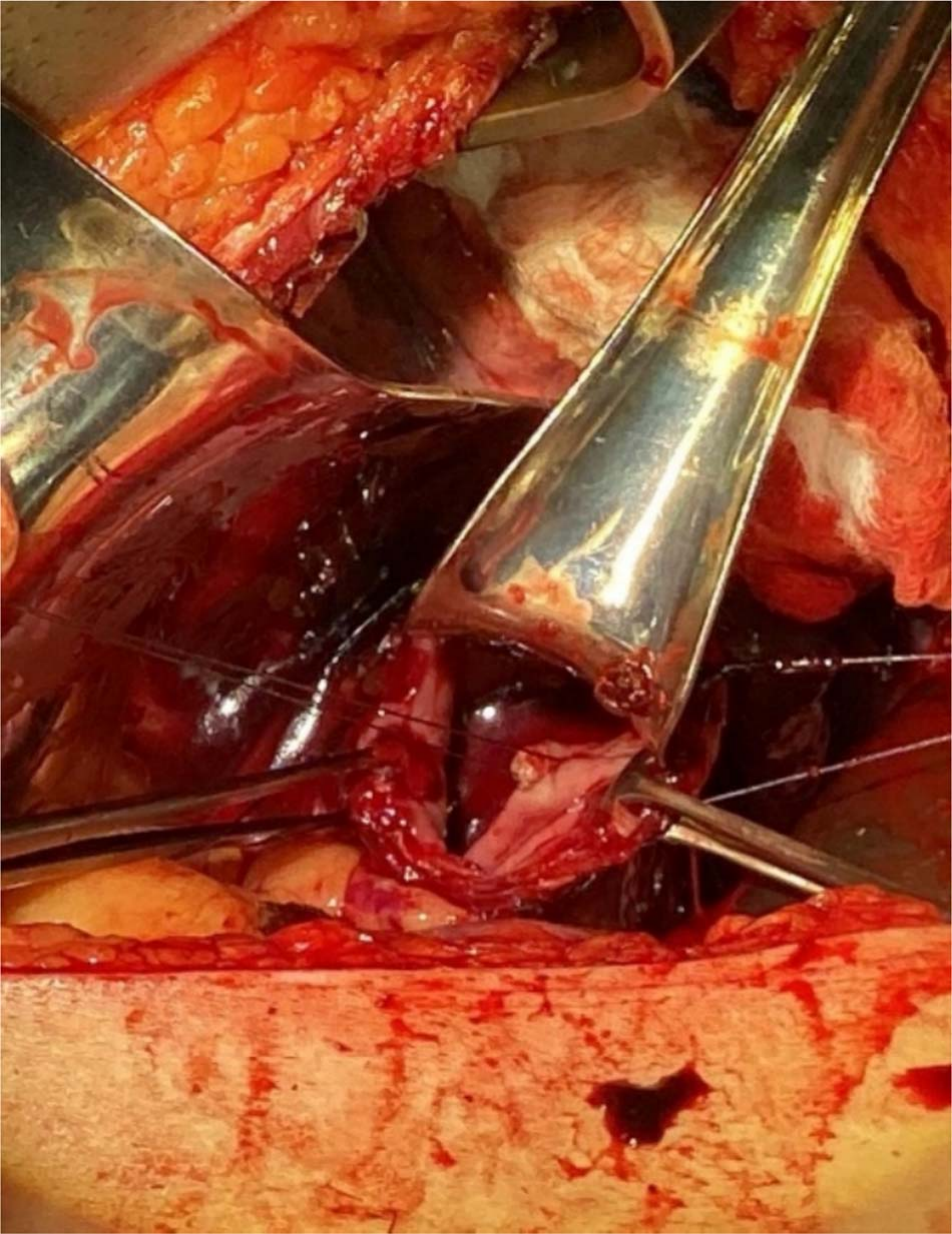
Illustrates the oversewing of the bleeding vessel to achieve hemostasis.

Surgical intervention was undertaken the day after admission. Both laparotomy and right anterolateral thoracotomy were performed. Intraoperative findings included markedly dilated and sigmoid-shaped esophagus, containing blood and residual food material. The initial surgical step involved mobilizing the esophagus from the hiatus, followed by a distal esophagotomy. Upon inspection, an active pulsatile arterial bleed was identified on the right lateral wall of the distal third of the esophagus, consistent with a Dieulafoy lesion. The bleeding vessel was successfully oversewn to achieve hemostasis (Fig. 3).

At this point, a critical surgical decision had to be made regarding further management of the esophagus. Given that the esophagus was profoundly dilated and nonfunctional, esophagectomy was deemed the most appropriate course of action. The esophagectomy was performed; however, the patient's condition was not sufficiently stable to permit immediate reconstruction. A second-look surgery was planned.

In a planned second surgery, a gastric conduit was fashioned from the greater curvature. As attempts to advance it through the hiatus were unsuccessful due to tension, a substernal pull-up was performed instead. The reconstruction was completed with a cervical esophagogastrostomy and closure of the hiatus.

## Discussion

This case highlights the complexity of managing life-threatening esophageal bleeding in a patient with end-stage achalasia and previous surgical intervention. Achalasia, a primary esophageal motility disorder, often leads to progressive esophageal dilation, food retention, and eventually a nonfunctional esophagus if left untreated or if interventions fail [8, 9]. Our patient had a history of Heller myotomy with Nissen fundoplication, yet ultimately developed marked esophageal dilation and a sigmoid-shaped esophagus.

Acute hematemesis with hemodynamic collapse in patients with achalasia is exceedingly rare. The identification of a Dieulafoy lesion—a dilated, aberrant submucosal vessel prone to acute bleeding—as the source in the distal esophagus is remarkable. Dieulafoy lesions are far more commonly found in the stomach and rarely in the esophagus, with few reported cases, particularly in the setting of chronic esophageal stasis and inflammation as seen in long-standing achalasia. The literature does not establish a direct causal relationship between chronic esophageal stasis and inflammation due to long-standing achalasia and the development of esophageal Dieulafoy lesions. However, chronic mucosal irritation and inflammation, as seen in achalasia, are recognized risk factors for various vascular and mucosal abnormalities, and could theoretically predispose to Dieulafoy lesion formation, though this remains speculative and not well documented in the literature [8–10].

Endoscopic control of acute gastrointestinal bleeding is the first-line approach but can be challenging, as in our case. Even when accessible, endoscopic techniques may not succeed in controlling brisk arterial bleeding from a Dieulafoy lesion. When initial endoscopic and interventional radiology approaches fail, prompt surgical intervention is warranted. This approach is supported by the American Society for Gastrointestinal Endoscopy. The British Journal of Surgery review and the American College of Radiology also emphasize that surgical intervention is the definitive next step after failure of both endoscopic and angiographic therapies [11].

The surgical management demanded a staged, multidisciplinary approach due to the patient’s critical state. The specific surgical approach (e.g. esophagectomy, segmental resection, or direct vessel ligation) depends on the location and extent of the lesion. An initial priority in our case was hemorrhage control, achieved through direct oversewing of the bleeding vessel after esophagotomy. The intraoperative finding of a severely dilated, nonfunctional esophagus necessitated esophagectomy—a decision supported by evidence that patients with long-standing achalasia and a nonfunctional, massively dilated esophagus, esophagectomy may be required, as described in case reports and reviews of similar clinical contexts [12].

Reconstruction following esophagectomy is particularly challenging in unstable patients. The safest approach is often delayed or staged reconstruction. This strategy allows for initial control of hemorrhage and patient stabilization before undertaking the technically demanding and high-risk reconstruction [13].

Substernal transposition of the gastric tube offered a practical alternative, with the hiatus closed to reduce the risk of mediastinal herniation or gastric conduit migration. Cervical esophagogastrostomy, though technically demanding, is considered advantageous in this setting, facilitating monitoring for anastomotic complications and improving postoperative outcomes [14]. Technical challenges include creating a substernal tunnel without injuring mediastinal structures, ensuring adequate vascular supply to the gastric conduit, and preventing conduit redundancy or kinking. Closure of the native hiatus is critical to prevent herniation and mediastinal contamination. The main advantage of the substernal route is the ability to achieve a tension-free anastomosis in anatomically distorted or previously operated fields, with acceptable long-term functional outcomes [14].

The postoperative course was complicated by acute respiratory distress syndrome, vocal cord paralysis. Vocal cord paralysis following esophagectomy and substernal esophagogastrostomy is most commonly caused by injury to the recurrent laryngeal nerve during cervical dissection or conduit transposition, particularly in anatomically distorted fields from chronic achalasia or prior interventions. The risk is heightened in cases requiring extensive dissection, as in patients with megaesophagus or after failed endoscopic and radiologic interventions for bleeding Dieulafoy lesions [15].

This case underscores the importance of tailored, multidisciplinary management in complex esophageal emergencies, the need to recognize rare causes of catastrophic bleeding in achalasia, and the utility of staged reconstructive surgery in high-risk patients. It also highlights the spectrum of potential postoperative complications and the importance of vigilant surveillance and early rehabilitation interventions to optimize recovery.

## Conclusion

This case illustrates the successful management of a rare esophageal Dieulafoy lesion causing catastrophic bleeding in end-stage achalasia. When endoscopic and angiographic attempts fail in patients with altered anatomy, prompt surgical exploration is critical. Staged reconstruction, like a substernal gastric pull-up, is a viable strategy in unstable patients, but success depends on vigilant multidisciplinary care to manage postoperative complications.
